# The role of emotion in clinical decision making: an integrative literature review

**DOI:** 10.1186/s12909-017-1089-7

**Published:** 2017-12-15

**Authors:** Desirée Kozlowski, Marie Hutchinson, John Hurley, Joanne Rowley, Joanna Sutherland

**Affiliations:** 1 Discipline of Psychology, School of Health and Human Sciences, Southern Cross University, Hogbin Drive, Coffs Harbour, NSW Australia; 2 School of Health and Human Sciences, Southern Cross University, Hogbin Drive, Coffs Harbour, NSW Australia

**Keywords:** Emotion, Emotional intelligence, Clinical decision making, Clinical reasoning

## Abstract

**Background:**

Traditionally, clinical decision making has been perceived as a purely rational and cognitive process. Recently, a number of authors have linked emotional intelligence (EI) to clinical decision making (CDM) and calls have been made for an increased focus on EI skills for clinicians. The objective of this integrative literature review was to identify and synthesise the empirical evidence for a role of emotion in CDM.

**Methods:**

A systematic search of the bibliographic databases PubMed, PsychINFO, and CINAHL (EBSCO) was conducted to identify empirical studies of clinician populations. Search terms were focused to identify studies reporting clinician emotion OR clinician emotional intelligence OR emotional competence AND clinical decision making OR clinical reasoning.

**Results:**

Twenty three papers were retained for synthesis. These represented empirical work from qualitative, quantitative, and mixed-methods approaches and comprised work with a focus on experienced emotion and on skills associated with emotional intelligence. The studies examined nurses (10), physicians (7), occupational therapists (1), physiotherapists (1), mixed clinician samples (3), and unspecified infectious disease experts (1). We identified two main themes in the context of clinical decision making: the subjective experience of emotion; and, the application of emotion and cognition in CDM. Sub-themes under the subjective experience of emotion were: emotional response to contextual pressures; emotional responses to others; and, intentional exclusion of emotion from CDM. Under the application of emotion and cognition in CDM, sub-themes were: compassionate emotional labour – responsiveness to patient emotion within CDM; interdisciplinary tension regarding the significance and meaning of emotion in CDM; and, emotion and moral judgement.

**Conclusions:**

Clinicians’ experienced emotions can and do affect clinical decision making, although acknowledgement of that is far from universal. Importantly, this occurs in the in the absence of a clear theoretical framework and educational preparation may not reflect the importance of emotional competence to effective CDM.

**Electronic supplementary material:**

The online version of this article (10.1186/s12909-017-1089-7) contains supplementary material, which is available to authorized users.

## Background

In recent decades, the idea that emotion—affective states that have arousing or motivational properties—might play a role in decision making has been increasingly considered [1]. Research confirms emotions constitute a potent and pervasive driver of judgment and decision making processes. It is recognised that emotions influence risky decisions [2], reduce cognitive fixation [3], and enhance attention [4]. Conversely, when decisions involve conflict or anger, these emotions can compromise cognitive processing [4]. Moreover, it is recognised that emotional discomfort can lead individuals to reframe difficult problems through coherence shifting [5]. Emotion arising from a decision choice at hand, can lead to bias or overriding of rational action, and incidental emotions from one decision can carry over and influence decision in another similar situation [6]. Reflecting the growing recognition of the role of emotion in decision making, Keltner and Lerner proposed an emotion-imbued choice model of judgement and decision making [6]. This model postulates that whether emotions improve or degrade judgement and decision making depends on the interaction of cognitive and motivational mechanisms.

Clinical decisions are often made in contexts that are emotionally challenging and require clinicians to actively manager their own and others’ emotions. Recently, the authors of a review paper [7] concluded that emotion impacts patient safety, and urged healthcare professionals to have the courage to recognise that their emotions influence their practice. A review of the nursing literature [8] acknowledged a number of authors linking emotional intelligence (EI) to clinical decision making (CDM), suggestive that emotions may be integral to CDM. EI can best be understood as the ability to access and utilise emotional and cognitive information of self and others in order to inform behavioural responses [9]. Typically, EI abilities include having the awareness of emotions within self and others, regulating these emotions, and then integrating them into technical based knowledge [10, 11]. Some authors, for example, Freshwater and Stickley [12], imply that clinical decisions made without reference to emotions are mechanistic and inferior, while others [13] link social awareness with effective clinical practice. In accordant work, Bucknall [14] found that interpersonal relationships were one of the top three environmental factors influencing the quality of nurses’ CDM in critical care settings. This evidence is difficult to reconcile with the dominance of purely cognitive, rational and technical interpretations of CDM employed in models and theoretical frameworks.

While links have been made between emotions and CDM in the literature, theoretical models of CDM largely focus on the technical and cognitive aspects of reasoning and making decisions. Clinicians’ and others’ emotions are a largely overlooked factor in theoretical models and research on CDM [15–18]. Given CDM often occurs in emotionally challenging contexts, and can entail significant emotional labour [19] requiring clinicians to manage their own and others’ emotions, it would not seem unreasonable to expect theoretical models of CDM to acknowledge and incorporate a role for emotional capabilities in decision making processes.

To further understanding of the role of emotion in CDM, we undertook an integrative review of the literature. For the purposes of the review, clinical reasoning was defined as the thinking processes involved in evaluating and integrating available information within clinical contexts whereas CDM involves choosing among various alternatives to inform patient focussed and evidenced based diagnosis and behaviours. Thus clinical reasoning informs the endpoint of decision making [20].

### Models of clinical decision making

Traditionally, CDM has been perceived as a “hypothetico-deductive process of determining patients’ problems” [21], with attention given to how clinicians balance risk and make decisions. In this model, emotion is excluded from clinical reasoning and decision making, and the process of deriving hypotheses and estimating the probabilities of diagnostic “fit” follow a Bayesian or probability theory of judgement and decision making [22]. In the nursing literature three main models of CDM are recognised [23]; these are: “the information-processing model, the intuitive-humanist model and the cognitive continuum model” [23]. Banning [24] described the information-processing model of decision making—with its decision trees—as assuming a logical, rational processing of facts to reach a decision, and the intuitive-humanist model as evolving pattern recognition from novice to expert. Others [25] portray Hammond’s cognitive continuum theory—where strategies from ‘analytical’ through to ‘intuitive’ are employed according to the nature of the task—as a broad theory that recognises the fluid nature of the decision making process.

In his ‘Universal Model of Diagnostic Reasoning’ Croskerry [26] allowed for some influence of affective components in type 1—intuitive—clinical decisions, but not in type 2—analytical—decisions. More recently, Johansen and O’Brien [27] presented a new model for clinical decision making in nursing. The model was derived from their concept analysis of the nursing literature on clinical decision making, clinical judgement and problem solving. Although related concepts such as stress and situational awareness were included in the model, emotion as an explicit term was absent.

### Physiological evidence of emotional involvement in decision making

There is recent empirical evidence linking ‘intuitive decision making’ with galvanic skin response [28], a physiological indicator of autonomic arousal commonly associated with emotional state [29]. Payne [28] found that nurses demonstrating significantly more sympathetic activation while performing decision tasks in simulated medical emergency scenarios also performed better on the decision tasks. She [28] suggested that these results could be thought of as objective evidence of the use of intuition in nursing. Payne cited previous work [30, 31] on non-nursing decision making where participants’ increased physiological arousal was thought to facilitate decision making once they had acquired experience with choice tasks.

Bechara, Damasio, and Damasio [30] described the somatic marker hypothesis, a model of non-clinical decision making that highlights the role of what might be termed emotional learning and prediction. The idea incorporates the kind of pattern learning associated in the nursing literature with professional intuition. With prior experience of a certain scenario, when one encounters it again the learned association between actions previously taken, their consequences, and the subjective pleasurable or aversive outcome (i.e., emotional experience) produces an autonomic response, which is then used to make the present decision. The somatic marker hypothesis is underpinned by a range of consonant evidence for the role of the orbitofrontal cortex and the amygdala in decision making [30] and includes emotional arousal as a key influencing factor to decision making.

The role of experience was also highlighted in a recent brain imaging study [32] where participants, both interns (novices) and attending physicians (experts), were subjected to functional magnetic imaging (fMRI) while they undertook a clinical decision making task. Results yielded three patterns of neural activation: one common to both groups, one unique to novices, and the other unique to experts. Experts demonstrated a more diffuse pattern of activation, perhaps reflecting the greater experiences on which they had to draw. Interestingly, novices exhibited greater activation only in the ventral anterior cingulate cortex [32], a region associated with emotional processing [33].

In addition to the somatic marker hypothesis another decision making model relevant to this paper is that of the appraisal tendency framework (ATF) [34]. Within this model emotions are understood to have an active effect on both decisions and judgments. Emotional influences stemming from unrelated experiences to current decisions being made have been shown to be present, often out of the decision maker's awareness [35]. The ATF also incorporates a significant component of cognitive appraisals that differentiate emotional experiences and informs decision making through a harm/benefit analysis emerging from the social environment [36].

Traditionally clinical decision making has been perceived as a hypothetico-deductive process, yet there seems to be an association between emotions—and/or emotional competence—and clinical decision making. Current theories of clinical decision making may not sufficiently account for the role of emotions in decision making processes. Therefore, we sought to detail the experience of and behaviours directly related to emotion in the immediate context of clinical decision making.

### Aim

The aim of this review was to identify empirical evidence for the role of emotions and/or emotional intelligence in clinical reasoning and/or clinical decision making.

## Method

An integrative, systematic review method [37, 38], bringing together both qualitative and quantitative data, was used with the aim of providing a more complete understanding of the role of emotion in CDM [39]. Due to the diversity of the methodologies and heterogeneity of the included studies, we did not perform a meta-analysis. The literature search was intended to extract, from a large body of work, those studies that covered experienced emotion or emotional intelligence in the context of clinical reasoning and/or clinical decision making. Content analysis, a qualitative method of text based data analysis, was employed to derive a thematic understanding of the role of emotion in CDM that might guide future inquiry [40].

### Literature search and sources

The first author conducted a computerised search of the bibliographic databases PubMed, PsychINFO, and CINAHL (EBSCO). A health services librarian was consulted for the design of the search strategy. Key subject descriptors and MeSH terms were used to map terms to the database vocabularies. The search terms used were: emotions; emotional intelligence; emotion*; decision making, clinical; clinical decision making; and, clinical reasoning. Limiters were: scholarly journal, English language; and publication 2006-February 2017. Inclusion criteria were: 1) reports empirical data (qualitative, quantitative, or mixed-methods); 2) concerns clinicians in clinical settings; 3) concerns clinician emotion OR clinician emotional intelligence/emotional competence; 4) concerns the effect or influence or relationship between criterion 3 and clinical decision making OR clinical reasoning. The complete search strategy can be found in Additional file 1.

### Search outcomes

**Fig. 1:**
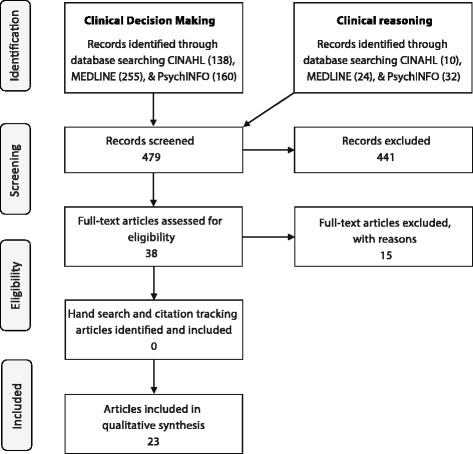
Overview of the literature search process

Following removal of duplicates, 479 studies were initially identified from the search strategy. Title and abstract screening was performed, with 38 papers retained. These retained papers were then reviewed at the full-text level. Fig. 1 illustrates the search progress via a PRISMA flowchart [41]. Full-text review identified a further 15 papers to exclude. Reasons for exclusion included dealing with a general or student sample rather than clinicians or not considering emotion in the immediate context of clinical decision making. Twenty three papers were retained for full analysis.

### Quality review

As no available tool was suited to evaluating the range of study designs included in this review, a modified set of appraisal criteria were derived by synthesising key criteria from pre-existing tools [42–45]. The design of the appraisal criteria allowed for comparability and consistency of the appraisal across study types. The tool contained eight generic items for evaluating the study design and methodology, against which all studies were appraised, and seven specific criteria for qualitative, quantitative and mixed methods studies. To conduct the quality review, members of the research team independently rated and scored each study according to the relevant criteria. Sum scores were created to allow comparison across studies, with high quality studies scoring >8, medium quality studies scoring 6-7, and low quality scoring <5. The results of the quality appraisal are summarised in Table 1. Data extraction and analysis.

**Table 1:** Quality review of retained papers

Criteria	1	2	3	4	5	6	7	8	9	10	11	12	13	14	15	16	17	18	19	20	21	22	23
All Studies																							
Theoretical model or framework evident	2	2	2	2	2	2	2	2	1	1	1	1	1	2	1	1	1	2	2	1	1	1	2
Question/objective sufficiently described	2	2	2	2	2	1	2	1	2	2	2	2	2	2	2	2	2	2	2	2	2	2	2
Inclusion/exclusion criteria are clearly defined	2	2	2	2	2	0	2	1	2	2	2	2	2	2	2	2	2	2	2	2	2	2	2
The study population is representative of population of interest	2	2	2	1	2	0	2	1	2	2		1	2	2	2	2	0	0	2	2	2	2	2
Accords with current ethical criteria, evidence of ethical approval	2	2		1	2	1	2	1	2	2	2	2	2	2	2	2	2	2	2	2	0	2	2
Results are reported in sufficient detail	2	2	2	1	2	1	2	1	2	2	2	2	2	2	2	2	2	2	2	2	2	2	2
Results are consistent with the data	2	2	2	1	2	1	2	1	2	2	2	2	2	2	2	2	0	2	2	2	2	2	2
Conclusions flow from the analysis or interpretation of the data	2	2	2	1	2	1	2	1	2	2	2	2	2	2	2	2	1	2	2	2	2	2	2
Quantitative Studies																							
Used random or probability sample												1									1		
Sample size adequate & representative including response rate												1									1		
Employed valid and reliable measures												2									2		
Confounding factors identified and managed												1									1		
Appropriate statistics employed												1									0		
Findings statistically or clinically significant												1									2		
Estimate of variance is reported for the main results												1									1		
Qualitative studies																							
Congruence between philosophical perspective and methodology	2	2	1		2	2	2	0	1	1	1		1	2	2	2	1	2	2			2	0
Influence of the researcher is addressed	1	1	1		1	2	2	0	1	1	1		2	1	1	2	1	2	2			2	2
Purposeful selection of participants, process clearly described	2	2	2		2	2	2	1	2	2	2		2	2	2	2	1	2	2			2	2
Congruence between research methodology & data collection	2	2	2		2	0	2	1	1	1	1		2	2	2	2	1	2	2			2	2
Congruence between research methodology & analysis methods	2	2	2		2	0	2	1	1	1	1		2	2	2	2	1	2	2			2	2
Use of audit or verification to establish credibility data analysis	0	0	0		0	2	2	1	1	1	1		2	2	2	2	1	1	2			2	2
Participants & their voices adequately represented	2	2	2		2	NA	2	1	2	2	2		2	2	2	2	1	2	2			2	2
Mixed Methods Studies																							
Mixed methods design is relevant to address the research question				1																2			
Influence of the researcher is addressed				2																2			
Adequate description of: methods, data handling, combing results				2																2			
Adequate integration of qualitative and quantitative results				1																2			
Total Score	27	27	24	17	27	15	30	14	24	24	22	22	28	29	28	29	17	27	30	23	21	29	28

Scoring: Yes = 2, No = 1, Not reported/unclear = 0; summary score was calculated for each paper by summing the total score obtained across relevant items. Adapted from M Hutchinson, L East, H Stasa and D Jackson [88], L Kmet, R Lee and L Cook [44], A Pearson [43], P Pluye, M-P Gagnon, F Griffiths and J Johnson-Lafleur [42]

Author details:

1 = Bach et al. 2009 [49]; 2 = Bryon et al. 2012 [50]; 3 = Calvin et al. 2007 [51]; 4 = Chaffey et al. 2010 [52]; 5 = Courtenay et al. 2009 [71]; 6 = Lafrance Robinson et al. 2015 [70]; 7 = Hov et al. 2009; 8 = McBee et al. 2015 [59]; 9 = Kim et al. 2016 [57]; 10 = McLemore et al. 2015 [60]; 11 = Smith et al. 2010 [62]; 12 = Alba 2016 [67]; 13 = Novick et al. 2015 [61]; 14 = Stolper et al. 2009a [63]; 15 = Stolper et al. 2009b [64]; 16 = Islam et al. 2015 [56]; 17 = Harun et al. 2015 [54]; 18 = Tentler et al. 2008 [66]; 19 = Tallentire et al. 2011 [65]; 20 = Alexander et al. 2014 [68]; 21 = Arevalo et al. 2013 [69]; 22 = Gallagher et al. 2015 [53]; 23 = McAndrew et al. 2015 [58]

The final manuscripts retained included quantitative, qualitative and mixed methods research and were suited to integrative synthesis [46]. To derive the synthesis, each article was reviewed independently by two authors with the main methods, themes, and findings of the study extracted and entered into a spreadsheet (see Table 2) [47]. After which, emergent themes were discussed within the team to derive consensus. Through a process of comparison, clustering and categorisation, the extracted findings were distilled across the studies, to derive the final two broad focal themes and sub-categories [48]. These were: i) those dealing directly with the experienced emotions of clinicians and how these emotions related to clinical reasoning and CDM; and ii), those examining skills related to emotional intelligence and clinical reasoning and CDM.

**Table 2:** Overview of the retained papers

First author, year	Country	Study design	Participant type (e.g., nurses) and number	Explicit CDM Model	Explicit Emotion/EI Model	Aspect/s of Decision making investigated
Alba, 2016 [67]	USA	Quantitative: correlational	Nurses (*n* = 182)	No	No	Ethical DM/intuition
Alexander et al., 2014 [68]	USA	Quantitative: descriptive	Physicians; Nurses (*n* = 71)	No	No	Evidence of Palliative Care providers’ compassionate response to patient emotional distress
Arevalo et al., 2013 [69]	Netherlands	Quantitative: cross-sectional	Nurses (*n* = 277)	No	No	Nursing role in end of life CDM
Bach et al., 2009 [49]	Canada	Qualitative: grounded theory	Nurses (*n* = 14)	No	No	Nursing role in end of life CDM
Bryon et al., 2012 [50]	Belgium	Qualitative: grounded theory	Nurses (*n* = 21)	No	No	Nurses’ CDM in relation to artificial nutrition or hydration for patients with dementia
Calvin et al., 2007 [51]	USA	Qualitative: Descriptive	Nurses (*n* = 12)	No	No	CDM during change of intensity of care/ end of life care
Chaffey et al., 2010 [52]	Australia	Qualitative: grounded theory	Occupational Therapists (*n* = 9)	Yes	No	Understanding and use of intuition in mental health practice
Courtenay et al., 2009 [71]	England	Mixed methods	Nurse prescribers (*n* = 40)	No	No	Communication; awareness of patient emotion in decision making
Gallagher et al., 2015 [53]	Brazil, UK, Germany, Ireland and Palestine	Qualitative: grounded theory	ICU nurses (*n* = 51)	No	No	ICU nurses’ end of life CDM practices in different cultural contexts
Harun et al., 2015 [54]	UK	Qualitative: thematic analysis	Dermatologists (*n* = 40)	No	No	Clinical and non-clinical influences on discharge decision making
Hov et al., 2009 [55]	Denmark	Qualitative: Phenomenology	Nurses (*n* = 14)	No	No	end of life CDM
Islam et al., 2015 [56]	USA	Qualitative: cognitive task analysis	Expert infectious disease physicians (*n* = 10)	Yes (proposed)	No	Cognitive mechanisms and complexity
Kim et al., 2016 [57]	Korea	Qualitative: content analysis	Nurses (*n* = 32)	No	No	Residential care CDM to preserve abilities in functional decline
Lafrance Robinson et al., 2015 [70]	Canada	Quantitative: cross-sectional survey	Clinicians from various disciplines (*n* = 305)	No	Iatragenic Maintenance Model & Therapist Drift Model	Negative influence of own and colleague’s emotions on CDM
McAndrew et al., 2015 [58]	USA	Qualitative: grounded theory	Nurses; Physicians (*n* = 7; 4)	No	No	end of life care
McBee et al., 2015 [59]	USA (army)	Mixed methods: experimental (IVs) and constant comparative approach	Physicians (*n* = 10)	Situated cognition	No	Effect of contextual factors on CDM
McLemore et al., 2015 [60]	USA	Qualitative: thematic analysis	Nurses (*n* = 25)	No	No	Abortion-related care, ethical CDM
Novick et al., 2015 [61]	Canada	Qualitative: constructivist grounded theory	Surgeons (*n* = 14)	No	No	Decision to call for expert assistance
Smith et al., 2010 [62]	Australia	Qualitative: hermeneutics	Physiotherapists (*n* = 14)	No	No	Effect of clinician experience on CDM in respiratory care
Stolper et al., 2009 [63]	Netherlands	Focus groups	GPs (*n* = 28)	Yes (proposed)	No	‘Gut feelings’ in diagnostic/prognostic processes
Stolper et al. B, 2009 [64]	Netherlands	Delphi consensus	GPs (*n* = 27)	No	No	Gathering consensus on ‘gut feelings’ in CDM
Tallentire et al., 2011 [65]	UK	Qualitative: grounded theory	Junior physicians (*n* = 36)	No	No	Factors affecting behaviour of newly qualified doctors
Tentler et al., 2008 [66]	USA	Qualitative: focus groups/RCT	Primary care physicians (*n* = 22)	No	No	doctors prescribing behaviours in response to patients’ requests for anti-depressants

## Results

The final papers included in the review all concern clinical reasoning and CDM by clinicians. As can be seen in Table 2, the final papers represented empirical work from qualitative [49–66], quantitative [67–70], and mixed-methods [71] approaches. They comprised of work with a focus on experienced emotion and on skills associated with emotions such as EI.

Of the retained studies, an explicit intention to examine both emotion and clinical reasoning/clinical decision making was expressed in the aims in only five [66–70], 12 set out to focus only on clinical reasoning/clinical decision making [49–54, 56–60, 62]. No studies specifically aimed to focus only on emotion. Six did not explicitly look at either EI or emotion, but these concepts are reported in the findings [55, 61, 63–65, 71]. The studies examined nurses (10), physicians (7), occupational therapists (1), physiotherapists (1), mixed clinician samples (3), and unspecified infectious disease experts (1). Two clear themes were identified, each having three distinct subthemes.

### The subjective experience of emotion in clinical decision making

Experienced emotion as an aspect of clinical reasoning and clinical decision making was highlighted in 14 studies [50, 51, 54–56, 58, 60–66, 70]. Three sub-themes emerged from the synthesis of findings reported in these studies: i) *emotional response to contextual pressures*, where clinical contexts of uncertainty, fear, conflict, discomfort, regret and unpredictability served as triggers for increased awareness of one’s own emotion; ii) *emotional responses to others*, where emotional self-awareness was sometimes described as a gut feeling that triggered the need for action; and, iii), *intentional exclusion of emotion from CDM*, where emotions were present in CDM, but mostly were not consciously foregrounded in decision making. Each of these themes are described in more detail below.

#### Emotional response to contextual pressures

Emotional responses of anxiety, stress and fear were evident where clinicians experienced pressures associated with CDM. In a qualitative study with infectious disease physicians (*n =* 10) in the USA, Islam, Weir, Jones, Del Fiol, and Samore [56] reported stress, complexity, and uncertainty were prominent experiences within the context of clinical reasoning. In this study, one of the three main themes identified was ‘social and emotional pressures’ within CDM. This comprised frustration and regret, liability and/or fear, and conflict around multiple care providers. The authors’ proposed model highlighted the relationship between cognitive mechanisms and social and emotional pressures acting on both type 1 and type 2 thinking processes. Stress and/or fear also featured in a grounded theory study on help-seeking behaviours by surgeons [61]. Calling in a second surgeon during procedures occurred for a range of both technical and non-technical reasons. Of relevance to this review, reasons for surgeons seeking help included being able to better manage their stress-related emotions around possible negative repercussions of decision making (“sharing the pain”) and to experience emotional support. Where this was done in a respectful and non-judgemental manner, positive experiences were reported. Tallentire, Smith, Skinner, and Cameron [65] also acknowledged the emotional contexts of decision making as including the need to manage stress and anxiety emerging from uncertainty in their grounded theory study of junior doctors. In this study, participants spoke of their reluctance to make decisions sometimes stemming from the conviction that causing harm by omitting treatment was somehow superior to causing harm by offering the wrong treatment. Tallentire et al. [65] suggested that emotional skills training should be part of medical education.

In the studies incorporated within this theme, two studies highlighted the presence of emotion within decision making specifically and the clinical context more widely [63, 64]. In these studies, emotions were initially denied or down played. Both framed these responses as “gut reactions”. In these studies emotional self-awareness was interpreted at the level of autonomic nervous system responses. Emotional awareness of the self was described in terms of gut reactions in the first paper [64]. This Delphi consensus study explored the sense of alarm/sense of reassurence reactions of 27 influential medical doctors across two countries in Europe. A sense of alarm described by participants as being reflective of an anxiety-based sympathetic nervous system alarm response. This response was identified as a key trigger to decision making that initiated further diagnostic exploration. The role of anxiety within CDM was also evident. The interplay of emotion and rational thinking was equally evident in further findings with additional focus groups of GPs exploring the role of gut feelings [63]. Participants described these gut sensations as extremely rapid signals that provided information “before they even started reasoning” (p. 20). The authors concluded that the emotional and rational characteristics of the GP were important factors in diagnostically using gut sensations. In turn, the likelihood that a GP would act on their gut sensation was itself thought to be sometimes influenced by emotion, in this case, fear of the opinion of colleagues.

#### Emotional response to others

The presence of emotional responses to others within everyday clinical decisions was evident in a focus group based qualitative study of 22 primary care physicians’ decision making around patient requests for anti-depressants [66]. Here, Tentler et al. [66] proposed a model where patients’ requests triggered both affective responses and cognitive responses in the physicians. These responses, mediated by various other influences, resulted in physicians employing either script-driven (utilising intuition or heuristics) or deliberative (deliberate, rational) reasoning. Annoyance and empathy were the primary emotional responses reported in this study, with around one third of the physicians acknowledging that their clinical judgement had been skewed by their reaction to the nature of the patients’ request. Of particular note, some physicians required facilitation to be made aware of this emotional aspect of their response to patients, after initially denying they were influenced. Tentler et al. [66] were not the only researchers to report findings along these lines. Harun et al. [54] conducted a qualitative interview-based study of 40 dermatology physicians’ decision making when discharging patients. Their data suggested that physicians’ decision making around discharge was at times biased by their emotions and gut feelings. Here again though, most clinicians were confident that such bias did not exist in their clinical reasoning and decision making.

From the nursing literature, Bryon et al. [50] interviewed nurses to report on their subjective experiences caring for patients with dementia, specifically around clinical decisions concerning artificial nutrition or hydration. The authors reported that the experience of ‘being touched’ by the vulnerability of their patients affected nurses’ own emotions, which, in turn, affected their care for the patients. ‘Being touched’ was interpreted as both positive and negative depending on the context, and empathy was a main theme of their findings. Vulnerability was also a theme in a paper by Hov et al. [55], but this time it was the nurses feeling vulnerable. The authors [55] conducted their hermeneutic phenomenological study with nurses caring for patients nearing the end of life (EOL) at nursing homes and found that nurses’ experienced emotions certainly influenced their CDM. For example, some nurses described anxiety around dying patients early in their career leading them to support maintaining treatment.

Calvin et al. [51], in their qualitative descriptive study of 12 nurse’s decision making in a neurological intensive care EOL context, outlined nursing and medical tensions around CDM with power differentials evident. In that study, nurses acted as the catalyst for family inclusion in CDM and arbitrated between family and medical officers; skills in emotional management of others underpinned that activity. The only other paper to directly address nurses’ subjective experience of emotion in relation to CDM was by McLemore et al. [60] who interviewed 25 nurses in abortion-related care. Their interviewees spoke richly of emotional reactions in their work and of the need to be self-aware and manage these emotions effectively. Skills such as these are associated with emotional intelligence, which we shall explore further below.

#### Intentional exclusion of emotion from CDM

Three studies reported intentional exclusion by clinicians of emotion from CDM [50, 55, 69]. In their grounded theory study of 11 nurses and physicians, McAndrew and Leske [58] found that, despite nurses’ and physicians’ emotional responsiveness to the human condition and feeling a connection with patients, nurses and physicians described keeping their emotions separated from their professional actions as important. The thematic analysis acknowledged emotional self-awareness was an important consideration in end-of-life decisions, but clinicians largely aimed to separate emotion from CDM. Similarly, in their study of cardiorespiratory physiotherapists, Smith et al. [62] reported that practitioners with more experience were better able to identify and manage their own emotions. The authors argued that this allowed them to separate their emotional responses from the clinical situation for beneficial outcomes [62]. Lafrance Robinson and Kosmerly [70] found that clinicians from various disciplines were significantly more likely to endorse the idea that emotions negatively affected the clinical decisions of *others* than their own decisions. Indeed, 40% of their participants agreed that ‘other’ clinicians experienced a negative effect on their clinical decisions as a result of emotion, while only 21.1% thought that the same applied to themselves.

### The application of emotion and cognition in CDM

The complex emotional contexts in which clinical decision making takes place as well as clinicians’ experiences, responses, and emotional/cognitive strategies for dealing with such environments were apparent in a number of the reviewed papers. Three sub-themes emerged from the synthesis: i) *Compassionate emotional labour – responsiveness to patient emotion within CDM, ii) Interdisciplinary tension regarding the significance and meaning of emotion in CDM, and iii) Emotion and moral judgement.* These themes foreground thoughtful integration of emotion and cognition and emotional awareness and emotional management of self and others, along with the skills and behaviours associated with clinicians’ emotional intelligence that were influential in clinical decision making.

#### Compassionate emotional labour – Responsiveness to patient emotion within CDM

Alexander et al. [68] analysed recordings of a range of palliative care clinicians’ consultations with patients and found that two thirds of conversations contained at least one expression of emotional distress. They found that clinician expressions of compassion in response to distressing emotions from patients and families were common (75.7%), with fewer than 2% ignored. Emotional expression was reported to be used at an appropriate time and in the right way to address issues causing patients emotional distress, clinical skills consistent with applied emotional cognition. Supporting palliative care patients in decision making was reflective of clinicians’ ability to use emotional reasoning and there was evidence of skills in the emotional management of others in that clinicians’ responses were appropriate to the issues causing patient emotional distress. Initiation of continuous palliative sedation was recognised as an event charged with emotion for nurses in a cross-sectional study [69]. Kim et al**.** [57]**,** in an interview based study of 32 nursing home nurses, included emotion in two of their five main themes related to managing residents’ remaining abilities. They found that CDM was undertaken through holistic cognitive dominated assessment that relied on the underpinning emotional awareness of others. CDM triggered holistic interventions across a physical-emotional spectrum with the inclusion of the emotional context of the patient leading to emotional interventions through emotional reasoning behaviours by the nurses**.** Gallagher et al. [53] sought better understanding of ICU nurses’ EOL decision making practices in different cultural contexts including Brazil, UK, Germany, Ireland, and Palestine. Skills in emotional reasoning and emotional management of others were utilised by the nurses to shift the focus of care away from curing and toward patient and family comfort and support.

In their qualitative study of nurses in abortion care, McLemore et al. [60] highlighted moral distress around ethical aspects of CDM. Nurses employed their personal experiences in this field as a foundation for empathy toward women. Within emotionally and ethically challenging contexts, CDM required real time and ongoing insight into personal emotions and the ability to manage these emotions during decision making. Again, although the authors did not explicitly interpret the study findings as EI, they noted that nurses integrated awareness of self and others into their clinical decision making. These capabilities allowed nurses to access personal emotions and then manage these emotions. This was undertaken simultaneously with the application of professional responsibilities and technical knowledge during decision making. This in itself is indicative of emotional reasoning, although not specifically articulated as such by the authors. Authentic communication was also highlighted in this study as a component of CDM [60], with study participants describing capabilities of effective emotional expression and emotional reasoning informed by emotional awareness of others as triggers for patient based CDM and consequent behaviours.

Gallagher et al. [53] further highlighted CDM behaviours that were underpinned by emotional self-management among nurses as they engaged in compassionate care. Also examining nurses’ CDM within end of life contexts, Bach et al. [49] similarly identified compassion and a desire to engage in authentic communication with patients and family. Though the authors identified emotional labour within this process, and noted the need for clinician self-awareness, they do not explicitly identify EI capabilities within this process. Similarly, Gallagher et al. [53], foregrounded self-awareness in attending and being fully present with patients. Although not explicit in the paper, it seems plausible that EI capabilities, such as emotional awareness of self and others and emotional management and reasoning, are likely to inform this process.

#### Interdisciplinary tension regarding the significance and meaning of emotion in CDM

Tensions around power differentials between nursing and medical staff in CDM were evident in the grounded theory study of nurses’ EOL decision making by Bach, Ploeg, and Black [49]. However, nurse motivation triggered by emotional awareness of others and resultant emerging compassion to initiate and engage in authentic communication (emotional expression) enabled active patient and family participation in CDM. Bryon and colleagues’ [50] grounded theory study of Flemish nurses, also highlighted nursing and medical divergence around CDM based on medical opinion rather than patient holistic needs. The authors suggested that empathy and compassion for patients acted as powerful motivation to provide holistically appropriate care interventions. Again, though not clearly articulated by the authors, evident in the study were the capabilities of emotional expression and emotional reasoning. Also in the study, authentic communication was characterised by appropriate emotional expression. Courtenay et al. [71] touched on a medical/nursing divergence too, with both nurses and doctors reporting that nurse prescribers spent more time talking about patient emotions during the consultation. Both reported that they believed nurses had a different style of consultation, in that they spent more time listening and offering support to the patient. The relationship developed with patients was found to be central to nurses’ prescribing decisions. While not overtly stated, from this study one could infer that applied emotional cognition plays a part in the CDM prescribing process. Chaffey et al. [52] also undertook a grounded theory study, this time of nine occupational therapists’ intuition-based CDM. The authors proposed a grounded theory of intuition in occupational therapy, where emotional understanding is considered a key component of intuition. The authors posited that emotional self-awareness and emotional reasoning were employed to clarify intuition and gut feelings.

#### Emotion and moral judgement

Factors related to emotional intelligence in CDM have also been explored through quantitative and mixed methods approaches. For example, Alba [67] measured nurses’ capacity and reliance on both rational and experiential thought processes as well as their ethical CDM skills. She found a small but significant correlation between experiential processes—which comprise feelings, emotions, and intuition—and ethical decision making and wrote “emotions are the significant driving force in moral judgement” (p. 9).

## Discussion

Despite the absence of emotion from accepted theoretical models of CDM, this review and synthesis of the empirical evidence found sufficient evidence to conclude that clinicians’ experienced emotions and their perceptions and understanding of others’ emotions can and do influence their decisions. We identified two main themes in the context of clinical decision making: the subjective experience of emotion; and, the application of emotion and cognition in CDM. Sub-themes under the subjective experience of emotion were: emotional response to contextual pressures; emotional responses to others; and, intentional exclusion of emotion from CDM. Under the application of emotion and cognition in CDM, sub-themes were: compassionate emotional labour – responsiveness to patient emotion within CDM; interdisciplinary tension regarding the significance and meaning of emotion in CDM; and, emotion and moral judgement.

Medical oriented qualitative studies identified here have an underlying theme of anxiety and stress associated with health care provision and this consciously or unconsciously impacting on clinicians and, hence, on the decisions they make and on how they communicate those decisions. Uncertainty is a key feature of the emotional context of CDM and in medical practice has been strongly associated with stress and fear [72] with denial being a common defence mechanism [73]. From a research perspective there may be value in further exploring inter-disciplinary and/or team based approaches to these emotional contexts of medical CDM. Such approaches appear to indicate higher reliability and hence diminished uncertainty [74].

Additionally, the medical literature has emotional CDM in the background to procedural based CDM, while nursing literature more strongly figures the emotional and patient advocacy aspects of nursing CDM, as well as wider nursing roles. Although bodily sensations described as gut feelings could be considered a kind of emotional awareness triggering the need for adjustment or action [63], there is also evidence of a tendency for physicians to allow the emotional content of clinical situations to go unacknowledged [54, 66]. Indeed, one study was excluded from this review at the full text stage because, although the authors purported to examine the impact of physicians’ “affect” on their CDM, the study did not explicitly report on clinician emotion at all [75]. End of life and aged care nursing literature was especially prominent with the application of emotion influenced CDM.

The acknowledgement and mastery of the emotional aspect of clinical decision making has been suggested as a critical element to improving patient safety [76]. Yet this review suggests there is a high level of variability in the integration of emotional awareness and competence with the cognitive aspects in CDM. As Alba [67] points out, experience does not always equate with expertise. Emotional Intelligence (EI) can be thought of as a set of emotional competencies compatible with this kind of emotionally-informed cognition and it is possible to increase EI [77]. Indeed, Chabeli [78] suggested that critical thinking builds emotional intelligence and Pichoff et al. [79] propose a link between EI and patient safety.

The broad construct of EI is concerned with the effective interaction of accessing and utilising both emotion and cognition to inform behavioural choices [80]. However, motivation is integral to whether an individual chooses to use their EI within CDM to inform their clinical actions or not [23]. This review suggests fertile ground in which to build the need and hence motivation to do so and reflects other authors [81, 82] reporting the relevance of EI to CDM.

However, this review identified only 5 studies where a consideration of emotion and CDM was an explicit aim. The other retained papers looked at one or other of these elements, or covered them incidentally. This represents a great opportunity for further work on the role of emotion and EI in CDM. The paucity of focus on emotion in empirical work is equally apparent in theoretical models of CDM. Alongside studies intended to explicate more fully the role of emotion and EI in CDM, development of a model of CDM that integrates recognition of the true role/s of emotion and EI skills is needed. We are not the first to call for this, for example, Alba [67] wrote “rational models do not capture the emotion and reality of human choice” (p. 1). Opportunities exist too in development of EI curricula in clinical education, training, and continuing professional development as well as clinical incident review and quality improvement.

Three opportunities have been identified through this review of primary evidence. Firstly, research designed to provide a more thorough and intentional picture of the ways emotion influences CDM is called for. Secondly, theoretical models of clinical decision making could become more nuanced and externally valid if they incorporated emotional aspects more fully. Finally, a focus on building emotional capabilities in our clinicians, both pre- and post-registration, may be an effective step toward increasing patient safety as well as clinicians’ feelings of self-efficacy. EI appears to be a construct to build such emotional capabilities as it is already associated with improved outcomes in work wellness [83], lower stress [84], retention [85], interdisciplinary teamwork [86], clinical teaching effectiveness [87], and overall clinical performance [85].

## Conclusion

Clinicians’ experienced emotions can and do affect clinical decision making, although acknowledgement of that is far from universal. We found ample evidence, albeit of variable quality, to confirm that both emotion and cognition are engaged in clinical decision making. Importantly, this occurs in the in the absence of a clear theoretical framework and educational preparation may not reflect the importance of emotional competence to effective CDM.

## Additional file

Additional file 1: Search strategy CINAHL. Details of the search strategy used for CINAHL. The search strategy was appropriately tailored for the other two databases searched. (DOCX 17 kb)

## Abbreviations

ATF: Appraisal tendency framework
CDM: Clinical decision making
EI: Emotional intelligence
EOL: End of life
fMRI: functional magnetic resonance imaging
GP: General practitioner
ICU: Intensive care unit
IV: Independent variable
MeSH: Medical subject headings
PRISMA: Preferred Reporting Items for Systematic Reviews and Meta-Analyses
USA: United States of America
