# Supplementary material for: The role of emotion in clinical decision making: an integrative literature review
Source: BMC Med Educ. 2017 Dec 15;17:255. doi: 10.1186/s12909-017-1089-7 (PMC5732402; doi:10.1186/s12909-017-1089-7)
Supplement: Additional file 1: — Search strategy CINAHL. Details of the search strategy used for CINAHL. The search strategy was appropriately tailored for the other two databases searched. (DOCX 17 kb) [file 12909_2017_1089_MOESM1_ESM.docx]

**Additional File 1: Search strategy CINAHL^[[1]](#footnote-1)^**

Search modes – Boolean/Phrase

| **Topic** | **Index terms and keywords** |
| --- | --- |
| Emotions | S1 Emotions OR emotional intelligence |
| Emotional intelligence (has a note) | S2 emotional intelligence (keywords) |
|  | S3 “emotional intelligence” Or emotion* |
| Decision making, clinical (The rendering of a judgment about patient care using analytical and intuitive processes and incorporating professional knowledge. Consider also DIAGNOSTIC REASONING and CRITICAL THINKING). | S4 Decision making, Clinical |
|  | S5 Clinical decision making |
|  | S6 Clinical reasoning (keyword – no heading listed) |
| Combined search with delimiters (Limiters Scholarly peer reviewed | S7 S1 OR S3 |
|  | S8 S4 OR S5 |
|  | S9 OR S7 |
|  | S10 S6 AND S7 |

**Medline**

Subject headings:

**Clinical Decision-Making**

**Emotional Intelligence**

**Emotions**

Limiters: scholarly journal; 2006-2017

**PsychINFO**

Subject headings:

**Clinical Decision-Making**

**Emotional Intelligence**

**Emotions**

Limiters: scholarly journal; 2006-2017

**Inclusion criteria:**

1. Article in English

AND

2. Published in a scholarly journal

AND

3. Publication date between 2006 and 2017

AND

4. Concerns clinician emotion OR clinician emotional competence/EI

AND

5. Concerns effect/influence of 4 on clinical decision-making OR on clinical reasoning

AND

6. Reports empirical data (qual or quant) Clinicians/clinical settings

1. The search strategy was appropriately tailored for the other two databases searched. [↑](#footnote-ref-1)
